# Survey dataset on occupational stress, job satisfaction, and job performance among male fertility patients

**DOI:** 10.1016/j.dib.2024.110152

**Published:** 2024-02-05

**Authors:** Nur Fatma Husna Majid, Suriyani Muhamad, Suhal Kusairi, Roszaman Ramli

**Affiliations:** a Faculty of Business, Economics and Social Development, Universiti Malaysia Terengganu, Terengganu, Malaysia; b Faculty of Economics and Business, Telkom University, Bandung, Indonesia; c Faculty of Medicine and Defence Health, Universiti Pertahanan Nasional Malaysia, Kuala Lumpur, Malaysia

**Keywords:** Job stress, Job satisfaction, Job performance, Presenteeism, Semen analysis, Sperm quality

## Abstract

This article presents data on occupational stress, job satisfaction, and job performance among male fertility patients. The data were collected from 11 November 2021 until 30 October 2022. A quantitative research approach was employed, involving a questionnaire development and survey. A sample of 309 was selected, using simple random sampling, from the pool of male patients that had undergone seminal fluid analysis (SFA) and received treatment from four private fertility clinics in Malaysia. Respondents were asked to give their consent by signing a consent form, for ethical research purposes. The questionnaire asked respondents about their demographics, sperm quality, occupational stress, job satisfaction, and job performance. The data could help other researchers to develop research on management issue in the context of male fertility, as well as organisations to maintain the health and welfare of their employees.

Specifications Table

**Table:** 

Subject	Management
Specific subject area	Organizational behaviour, human resource management and health economics
Data format	Raw, descriptive data
Type of data	Table, Raw data (.xlsx)
Data collection	Simple random sampling was used to select a sample from which data were to be collected. Respondents were male patients of private clinics in Malaysia, with normal and abnormal sperm quality (i.e. oligozoospermia, asthenozoospermia, teratozoospermia, oligoasthenozoospermia, asthenoteratozoospermia, oligoasthenoteratozoospermia and azoospermia). Data were collected using a questionnaire developed based on Management Standards Indicator Tool [1], work-family conflict [2], Job Satisfaction Survey [3], and Standford Presenteeism Scale [4,5]. Respondents were given information about the survey, and data were gathered using paper and pencil method. Respondents were instructed to complete the survey right away and turn in the questionnaires at the end of the session. Clear and detailed instructions were provided to avoid a misunderstanding or improper responses by respondents. Furthermore, respondents were guided when answering the questions in order to minimise errors.
Data source location	Institution: Private fertility clinic City/Town/Region: Terengganu, Pahang, Kuala Lumpur Country: Malaysia
Data accessibility	Repository name: Mendeley Data Data identification number: 10.17632/j762xjdpmr.1 Direct URL to data: https://data.mendeley.com/datasets/j762xjdpmr/1

## 1 Value of the Data

• The dataset may be utilised to examine correlations between different levels of occupational stress and varying aspects of job performance among male fertility patients.
• The data may be used to analyse how different demographic factors, such as age, marital status, or educational level interact with job satisfaction in this specific group.
• This data may contribute to the understanding of how occupational stress and job satisfaction influence presenteeism among male fertility patients, potentially affecting organisational productivity.
• This dataset increases the limited literature on issues regarding job performance among male fertility patients in Malaysia and helps to broaden studies on the subject.
• The data may provide an insight to increase public understanding of issues regarding job performance and male fertility patients and serve as a reference for organisations to improve the working environment and promote awareness on the issues.
• The dataset may offer a comparative perspective for studies in countries with similar culture and economic dynamics or with different healthcare systems or work cultures to provide a richer and more diverse understanding of the interplay between job-related factors and male fertility issues.

## 2 Background

Individual job performance is an issue that has not only attracted the attention of organisations worldwide but has also stimulated extensive research in management, occupational health and organisational behavior, and organisational psychology [6]. Occupational stress and job satisfaction are often mentioned when discussing job performance as these variables are interrelated to each other [7], [8], [9]. Workers that rated themselves as doing poorly on job performance were reported to have high occupational stress and low job satisfaction. A study found that men are less productive at work as they struggle to concentrate due to infertility treatment [10]. However, the existing literature does not go into great detail about how men feel about the link between their work and infertility [11]. The dataset is aimed at helping researchers gain a greater understand of the relationship between occupational stress, job satisfaction, and job performance among male fertility patients.

## 3 Data Description

This article provides data on demographics (Table 1), sperm quality (Table 2), and a descriptive analysis of occupational stress, job satisfaction, and job performance (Table 4). The demographic characteristics presented in Table 1 include age, religion, ethnicity, marital status, marriage period, number of children, educational level, employment status, working period, and household income.

**Table 1:** Demographic characteristics of respondents.

Demographic Characteristics	Items	Frequency	Percentage (%)
Age	29 years and below	28	9.1
	30–39	208	67.3
	40–49	68	22.0
	50–59	4	1.3
	60 years and above	1	.3
Religion	Islam	297	96.1
	Christian	1	.3
	Buddha	8	2.6
	Hindu	3	1.0
Ethnicity	Malays	291	94.2
	Bumiputera	3	1.0
	Chinese	10	3.2
	Indians	3	1.0
	Other	2	.6
Marital status	First marriage	278	90.0
	Second marriage or more	31	10.0
Marriage period	5 years and below	112	36.2
	6–10	121	39.2
	11 years and above	76	24.6
No. of children	No children	223	72.2
	1–2	72	23.2
	3 or more	14	4.5
Educational level	Did not complete primary school	1	.3
	Completed Standard 6	2	.6
	Completed Form 3	14	4.5
	Completed Form 5	93	30.1
	Completed Form 6/certificate/diploma	99	32.0
	Completed Degree	75	24.3
	Completed Masters	13	4.2
	Completed PhD	12	3.9
Employment status	Full-time	300	97.1
	Part-time	5	1.6
	Unemployed	4	1.3
Working period	<1 year	4	1.3
	1–3	10	3.2
	3–5	28	9.1
	5–10	73	23.6
	>10 years	194	62.8
Household income	<RM2,500	26	8.4
	RM2,500 - RM3,169	35	11.3
	RM3,170 - RM3,969	31	10.0
	RM3,970 - RM4,849	39	12.6
	RM4,850 - RM5,879	46	14.9
	RM5,880 - RM7,099	38	12.3
	RM7,110 - RM8,699	28	9.1
	RM8,700 - RM10,959	25	8.1
	RM10,960 - RM15,039	23	7.4
	RM15,040 or more	18	5.8

**Table 2:** Sperm quality of the respondents.

Type	Frequency	Percentage
Normal	144	46.6
Oligozoospermia	52	16.8
Asthenozoospermia	48	15.5
Teratozoospermia	1	.3
Oligoasthenozoospermia	24	7.8
Asthenoteratozoospermia	1	.3
Oligoasthenoteratozoospermia (OATs)	8	2.6
Azoospermia	31	10.0
Total	309	100.0

Table 2 highlights the frequency of the different types of sperm quality of the respondents. Normal sperm quality was observed in 144 respondents (46.6%) while abnormal sperm quality was detected in 165 respondents (53.4%). Table 3 shows the types of sperm quality and their descriptions according to World Health Organization [12]. The three main sperm parameters are concentration, motility and morphology. Azoospermia is defined as the complete lack of spermatozoa after centrifugation of sperm.

**Table 3:** Nomenclature related to sperm quality.

Type of sperm	Description
Oligozoospermia	Sperm concentration less than 15 million
Asthenozoospermia	Progressive motility percentage less than 32%
Teratozoospermia	Normal sperm morphology percentage less than 4%
Oligoasthenozoospermia	Sperm concentration and progressive motility less than lower reference limit
Asthenoteratozoospermia	Percentage of progressive motility and normal sperm morphology less than lower reference limit
Oligoasthenoteratozoospermia	Sperm concentration, progressive motility and sperm morphology of normal form less than lower reference limit
Azoopsermia	No spermatozoa in the ejaculate

Table 4 presents the descriptive analysis of the variables. The distribution of occupational stress was found to be left-skewed, with a skewness of −0.213. Occupational stress was found to have a kurtosis of −0.314, which indicates its distribution was light-tailed and not normal. In terms of job satisfaction, the measured skewness was −0.233, indicating a left-skewed distribution. Job satisfaction had a kurtosis of −0.179, indicating a light-tailed distribution instead of a normal one. Regarding job performance, calculated skewness was −0.322, signaling a left-skewed distribution. Job performance had a kurtosis of 0.117, indicating the variable was normally distributed. The raw dataset and codebook have been uploaded for sharing in Mendeley Data repository [13] in Excel format (DIB dataset and codebook.xlsx) and contains demographic information (age, religion, ethnicity, marital status, marriage period, number of children, level of education, employment status, working period and household income), sperm quality, occupational stress (OS1-OS41), job satisfaction (JS1-JS36), and job performance (JP1-JP6). A survey questionnaire in Malay, with English translation provided, has been appended as a supplementary document (DIB questionnaire.docx). All data were collected and shared in accordance with the ethical standard of Universiti Malaysia Terengganu, the private fertility clinics, and the Declaration of Helsinki. Specific measures were taken to ensure respondent confidentiality and data anonymisation. Their use should be strictly for academic and research purposes, in compliance with ethical research practices.

**Table 4:** Descriptive statistics of the variables.

Variable	N	Min	Max	Mean		Std. Deviation	Skewness		Kurtosis	
				Statistic	Std. Error		Statistic	Std. Error	Statistic	Std. Error
Occupational stress	309	2.61	4.76	3.8374	.02438	.42862	−0.213	.139	−0.314	.276
Job satisfaction	309	2.25	5.00	3.7146	.02975	.52289	−0.233	.139	−0.179	.276
Job performance	309	2.33	5.00	4.1936	.03051	.53637	−0.322	.139	.117	.276

## 4 Experimental Design, Materials and Methods

This research used a simple random sampling technique to select a sample and conduct a cross-sectional survey. Data were gathered from 309 male patients from four private fertility clinics in Terengganu, Pahang, and Kuala Lumpur, who had undergone seminal fluid analysis (SFA). The fertility clinics in Pahang and Terengganu are the referral centres for the east coast region which covers Pahang, Terengganu, and Kelantan. Meanwhile, the private fertility clinic in Kuala Lumpur covers the west coast area. With these two main areas covered, we were thus able to capture a more diverse demographic and socioeconomic background of the patients. The sample's various demographic characteristics represented those of the broader population of Peninsular Malaysia. Their semen samples were collected and examined according to World Health Organization criteria [12]. The four medical facilities were selected based on (1) fertility treatments provided including seminal fluid analysis (SFA) and (2) approval from the specialist doctors to involve their patients in this research. Only male patients who had signed the consent form were given a set of questionnaires to answer.

The questionnaire used close-ended questions from previous research, and these were back-translated into Malay. The questionnaire consisted of five sections, namely Section A: Sociodemographics, Section B: Occupational stress, Section C: Job satisfaction, Section D: Job performance, and Section E: Male fertility. Section A asked for respondents’ demographics. This section included questions about age, religion, ethnicity, marital status, marriage period, number of children, educational background, employment status, working period, and household income.

Section B combined items adapted from Management Standards indicator tool by Health and Safety Executive [1] and family-work and work-family conflict by Pejtersen et al. [2] to assess occupational stress (OS). The six areas of Management Standards indicator tool adapted were demand, control, relationships, change, role, and support, and the total number of items was thirty-five. Each indicator consisted of three to eight items with 5-point Likert scale responses, which were 1 “never”, 2 “seldom”, 3 “sometimes”, 4 “often” and 5 “always”. Work-family conflict had four items while family-work conflict had two items, both providing four-point Likert scale responses. Item 1 under work-family conflict used the following responses: 1 “yes, often”, 2 “yes, sometimes”, 3 “rarely” and 4 “no, never”. Meanwhile, item 2 until item 4 under work-family conflict and two items from family-work conflict used the following responses: 1 “yes, certainly”, 2 “yes, to a certain degree”, 3 “yes, but only very little” and 4 “no, not at all”. In total, Section B (Occupational Stress) consisted of forty-one items with positive- and negative-worded item. Some examples of OS items are as follows: “I have unachievable deadlines,” “I have choice in deciding how I do my work,” “Do you feel that your work drains so much of your energy that it has a negative effect on your private life?” “Do you feel that your private life takes so much of your time that it has a negative effect on your work?”

Section C consisted of thirty-six items adopted from Job Satisfaction Survey by Spector [3] and translated by Tan Soo Luan into Malay [14]. The section assessed nine factors of job satisfaction (JS), namely promotion, pay, benefits, supervision, nature of work, rewards, communication, operating procedures, and coworkers. Each aspect had four items with positive- and negative-worded items. All items had five-point Likert scale responses, with 1 being “strongly disagree”, 2 “disagree”, 3 “neutral”, 4 “agree” and 5 “strongly agree”. Examples of the item are as follows: “I feel I am being paid a fair amount for the work I do,” “There is really too little chance for promotion on my job,” “My supervisor shows too little interest in the feeling of subordinates.”

Section D used the concept of presenteeism adapted from the Standford Presenteeism Scale by Koopman et al. [4] and Mokhtar et al. [5] to evaluate job performance despite having a fertility problem, and it consisted of six items relating to completing work and avoiding distraction. Completing work had three positive-worded items that focused on achieving the outcome of the work. Meanwhile, avoiding distraction focused on avoidance of distraction in the process of doing work, and it had three negative-worded items. All six items provided the following responses: 1 “strongly disagree,” 2 “disagree,” 3 “neutral,” 4 “agree,” and 5 “strongly agree.” Among the items are “I can complete my tasks despite my fertility problem,” and “I find it difficult to manage work-related stress because of my fertility problem.”

Section E asked about the fertility status or sperm quality of the respondents, whether normal or abnormal. Abnormal fertility consists of oligozoospermia, asthenozoospermia, teratozoospermia, oligoasthenozoospermia, asthenoteratozoospermia, oligoasthenoteratozoospermia (OATs), and azoospermia. However, this section was not for the respondents to answer, but it was marked by the staff from the fertility clinics.

All negative-worded items in Section B, C, and D were reverse-coded in the Excel data file. A self-administered questionnaire survey using paper and pencil was used to gather the data. The background and purpose of the study were explained to the respondents. The fact that all the provided information would be treated with strict confidentiality and used solely for research was also explained to them. To avoid misunderstanding or incorrect answers by respondents, clear and precise instructions were provided. The respondents were also guided when answering questions to minimize errors. Data were extracted into an Excel format and analysed through IBM SPSS Statistics for frequency, descriptive analysis, and reliability test. Table 5 below displays the Cronbach's alpha value for each variable. Alpha value for OS is 0.904, JS is 0.927 and JP is 0.817.

**Table 5:** Cronbach's alpha value of the variables.

Variable	No. of questions	Cronbach's alpha
Occupational stress	41	.904
Job satisfaction	36	.927
Job performance	6	.817

## Limitations

Fertility issues are often associated with women, while there is limited discussion on this issue involving men. Since male fertility is still regarded as a sensitive and taboo issue in the community, many patients feel embarrassed to openly discuss this issue. Thus, it was difficult to obtain consent from male patients to participate in this survey.

Another limitation was that there are limited clinics with available data on male fertility. It was hard to find clinics that provide consultation and fertility treatment for men. Some of the clinics only have a few cases of male fertility. We also experienced difficulty in obtaining approval from doctors to conduct this study since patient information is confidential. Despite these limitations, we strongly believe that our findings provide valuable preliminary insights into this under-researched area.

## Ethics Statement

All procedures implemented during the study adhered to the ethical standards of Universiti Malaysia Terengganu (UMT/JKEPM/2021/82). All respondents involved in the study gave their informed consent. It was made clear to the respondents that the survey was anonymous. The research was carried out in accordance with the Declaration of Helsinki.

## CRediT authorship contribution statement

**Nur Fatma Husna Majid:** Writing – original draft. **Suriyani Muhamad:** Supervision, Writing – review & editing. **Suhal Kusairi:** Methodology, Formal analysis. **Roszaman Ramli:** Investigation, Resources.

## Data Availability

Dataset of occupational stress, job satisfaction and job performance of male fertility patients (Original data) (Mendeley Data).
